# NAD^+^ Precursors and Antioxidants for the Treatment of Amyotrophic Lateral Sclerosis

**DOI:** 10.3390/biomedicines9081000

**Published:** 2021-08-12

**Authors:** Elena Obrador, Rosario Salvador-Palmer, Rafael López-Blanch, Ryan W. Dellinger, José M. Estrela

**Affiliations:** 1 Department of Physiology, Faculty of Medicine, University of Valencia, 46010 Valencia, Spain; elena.obrador@uv.es (E.O.); rosario.salvador@uv.es (R.S.-P.); loblanch@alumni.uv.es (R.L.-B.); 2 Elysium Health Inc., New York, NY 10013, USA; ryan@elysiumhealth.com

**Keywords:** amyotrophic lateral sclerosis, motor neurons, oxidative stress, NAD^+^, antioxidants

## Abstract

Charcot first described amyotrophic lateral sclerosis (ALS) between 1865 and 1874 as a sporadic adult disease resulting from the idiopathic progressive degeneration of the motor neuronal system, resulting in rapid, progressive, and generalized muscle weakness and atrophy. There is no cure for ALS and no proven therapy to prevent it or reverse its course. There are two drugs specifically approved for the treatment of ALS, riluzol and edaravone, and many others have already been tested or are following clinical trials. However, at the present moment, we still cannot glimpse a true breakthrough in the treatment of this devastating disease. Nevertheless, our understanding of the pathophysiology of ALS is constantly growing. Based on this background, we know that oxidative stress, alterations in the NAD^+^-dependent metabolism and redox status, and abnormal mitochondrial dynamics and function in the motor neurons are at the core of the problem. Thus, different antioxidant molecules or NAD^+^ generators have been proposed for the therapy of ALS. This review analyzes these options not only in light of their use as individual molecules, but with special emphasis on their potential association, and even as part of broader combined multi-therapies.

## 1. Introduction

ALS, often referred to as “Lou Gehrig’s disease”, is a progressive neurodegenerative disease that damages motor neurons (MNs) in the brain and the spinal cord [1]. This loss of MNs is responsible for progressive weakness and paralysis. It is the third most prevalent neurodegenerative disease, after Alzheimer’s and Parkinson’s disease. It affects approximately 30,000 people in the U.S. and 450,000 worldwide [2]. ALS usually occurs in people over 50, although in a minority of cases it appears at younger ages. Men are slightly more likely to have ALS than women; however, as age increases, this difference disappears [3]. A recent study including approximately 3000 ALS patients concluded that the onset and progression of ALS is characterized by a different susceptibility of motor cortex and lower MNs to the ALS-associated damage, and is influenced by age, sex, and gene variants [4].

Although it is still considered a rare disease, approximately 20 people in the world are diagnosed with ALS every hour. Most cases of ALS (>90%) are considered sporadic (SALS), which means that the disease appears to occur randomly, without a clear family history of the disease. Only 5–10% of cases are associated with known genetic mutations (familial-type ALS, FALS) [1]. In patients with FALS, the symptoms usually appear earlier (approximately 5 years) than in those diagnosed with SALS, a fact apparently related to Mendelian gene variants lowering the age of onset [5]. The mean survival from the first symptom is 3–5 years, although approximately 10% of patients live longer than 10 years [6]. The reasons for this exceptional survival remain poorly understood.

Despite recent advances in the understanding of ALS, respiratory and nutritional interventions remain the treatments showing the highest efficacy in extending the patient’s survival. ALS has no effective treatment or cure to date [7].

There is mounting evidence involving oxidative stress as a main pathophysiological mechanism leading to MN damage and death [8]. Conceptually, oxidative stress is an imbalance of oxygen-derived free radicals and antioxidants in the body, which can lead to cell and organ damage [9]. Reactive oxygen species (ROS) contain an uneven number of electrons and, at high non-physiological levels, can cause oxidative damages to nucleic acids, lipids, and proteins [9]. Moreover, the generation of NO and H_2_O_2_ (induced by proinflammatory cytokines in e.g., endothelial cells) can lead to the formation of highly damaging ^–^OONO radicals [10,11]. Antioxidants are molecules that can donate an electron to a free radical without making themselves unstable. Therefore, enhancers of our physiological antioxidant defenses or the administration of molecules with direct antioxidant activity are potential therapeutic strategies against ALS [12,13]. 

On the other hand, NAD^+^ is involved in cell bioenergetics, redox regulation, signaling, homeostasis, adaptive response to stress, and survival [14,15]. Specifically, different NAD^+^-dependent enzymes are implicated in mechanisms regulating synaptic plasticity [16,17] and neuronal resilience to stress [18,19]. Therefore, either the inhibition of NAD^+^ consuming enzymes or the supplementation with NAD^+^ precursors also appears to be potentially useful in the therapy of ALS [20,21].

Oxidative stress and alterations in redox status, bioenergetics, and NAD^+^ metabolism are clearly linked to the pathophysiology of ALS [8]. Thus, the aim of this review is to discuss the possibility of using both NAD^+^ promoters and antioxidants as complementary therapies to slow down the progression of MN damage.

## 2. NAD^+^ and Physiological Antioxidant Levels in ALS

NAD^+^ is a coenzyme in redox reactions, a donor of ADP-ribose for ADP-ribosylation reactions, a precursor of cyclic ADP-ribose, and a substrate for sirtuins (SIRTs) that use the cofactor to remove acetyl groups from proteins. SIRTs link NAD^+^ levels to mitochondrial function, dynamics and biogenesis, and to cellular antioxidant defenses [8,22]. Interestingly, under the conditions of reduced cellular energy (as it occurs in the MNs during the progression of ALS), SIRTs may not consume sufficient NAD^+^ to preclude any cell survival-promoting effects of its deacetylase action on protein substrates [23]. For instance, poly(ADP-ribose) polymerase 1 (PARP1) is involved in DNA replication, transcription, DNA repair, apoptosis, and genome stability. However, DNA damage may overactivate PARP1 and lead to cell death and inflammation [24]. SIRT1 function (nucleus and cytoplasm) can facilitate cell survival under stress conditions by the deacetylation-dependent deactivation of PARP1 [25]. However, SIRT1 also consumes NAD^+^. Thus, inducing an increase in SIRT levels and activity (at least above an undefined threshold) could be counterproductive to neuronal survival, particularly to those energetically compromised. As an example, the nicotinamide-induced inhibition of SIRT1 was shown to protect neurons from death under acute anoxic injury [26]. Moreover, in SIRT1 knockout mice, their brains showed low levels of oxidative stress-related markers (i.e., carbonylated proteins and isoprostanes) [27]. These facts suggest a delicate balance between SIRT1 activity and the survival of neurons subjected to stress-associated insults. Despite the controversial facts regarding the activity of SIRT1, it is widely accepted that the maintenance of high NAD^+^ levels promotes cell homeostasis and survival [28]. Consequently, the consumption of NAD^+^ without an adequate method of replenishment is deleterious for the cell physiology.

In mammalian cells, NAD^+^ can be synthesized de novo from tryptophan (a multi-step enzymatic process which is fairly inefficient), or from nicotinic acid, nicotinamide (NAM), nicotinamide mononucleotide (NMN), or nicotinamide riboside (NR) [29,30]. For instance, it has been shown that NAM prevents NAD^+^ depletion and protects neurons against excitotoxicity and cerebral ischemia [31]. NR and NMN were shown to confer axonal protection in a Wallerian degeneration model of neuronal explant cultures [32]. Interestingly, the mechanism that neurons undergoing axonal degeneration use for protection is to upregulate the expression of nicotinamide riboside kinase (NRK) and nicotinamide mononucleotide adenylyl transferase 1 (NNMAT1), the enzymes required to convert NR (or NMN) to NAD^+^ [32]. Further, NR rescues mitochondrial defects and neuronal loss in models of Parkinson’s diseases [33], or increasing cytosolic and mitochondrial NAD^+^ content in ALS astrocytes increases the oxidative stress resistance and reverts their toxicity towards MNs [34]. Furthermore, it has been shown that SARM1 (sterile α and TIR motif–containing 1) is a key factor in triggering axon degeneration after an injury. Once activated in neurons, SARM1, which metabolizes NAD^+^ to NAM, depletes NAD^+^, leading to a massive loss of the energy supply within the axons [35]. These researchers were capable of reversing this detrimental effect by supplementing the neurons in which SARM1 was activated with NR [35].

NAD^+^ levels in mouse cortical neurons are of approximately 10 nmol/mg of protein [36], which is similar to those found in different types of neurons. The altered expression of enzymes involved in NAD^+^ synthesis (nicotinamide phosphoribosyltransferase and nicotinamide nucleotide adenyltransferase 2) and the decreased SIRT6 expression found in the spinal cord of ALS patients suggest deficits of this neuroprotective pathway in the human pathology [37]. Although precise in vivo data on NAD^+^ levels in the MNs of ALS models and patients are lacking, taking into account all available evidence, a significant NAD^+^ depletion is expected (see e.g., Park 2016) [38].

SIRT3, the main SIRT deacetylase in mitochondria, deacetylases and increases the activity of both superoxide dismutase 2 (SOD2) (thereby controlling the excess of toxic O_2_ [39]) and isocitrate dehydrogenase (which increases NADPH and, that way, favors the glutathione (GSH) reductase reaction) [40]. GSH is a main physiological antioxidant and the GSH/glutathione disulfide (GSSG) ratio is a measure of cellular oxidative stress [41]. Oxidation of this tripeptide, which cannot be synthesized inside the mitochondria and must be transported from the cytosol [42], facilitates the opening of the mitochondrial permeability transition pore and the release of proapoptotic death signals [43]. Importantly, early studies also demonstrated that GSH deficiency leads to mitochondrial damage in the brain [44].

There is evidence to suggest that the response to oxidative stress is dampened in ALS. GSH depletion promotes neurological deficits, mitochondrial dysfunction, and MN degeneration in mutant SOD1 ALS mice [45]. Levels of GSH are lower in the motor cortex of ALS patients as compared to healthy volunteers [46]. 

Consequently, this background strongly supports the notion of a close relationship between oxidative stress, NAD^+^ levels, cellular redox status, and mitochondrial function, all of which are involved in the pathophysiology of ALS [8]. 

## 3. NAD^+^ Promoters and Antioxidants to Protect Motor Neurons

### 3.1. NAD^+^ Promoters

NAD^+^ is a coenzyme that facilitates redox reactions and is found in all living cells. Potential NAD^+^ promoters now under study include (but are not limited to) niacin (NA), NAM, NMN, and NR [47,48,49,50]. 

NA is a water-soluble vitamin B3 that has been shown to increase tissue NAD^+^ in humans. In a recent clinical trial, patients with mitochondrial myopathy or a healthy age-matched control group were given a steadily increasing dose of NA, starting at 250 mg/day to 750–1000 mg/day over a 4-month period, and then a 10-month follow-up treatment period. NA treatment increased muscle NAD^+^ levels 1.3-fold at 4 months and 2.3-fold after 10 months in the study group. The control group saw no increase in NAD^+^ (NCT03973203, www.clinicaltrials.gov (accessed on 30 July 2021)) [51]. Nevertheless, the side effects and risks of taking high doses of NA are well known and include flushing, an upset stomach, diarrhea, liver damage, stomach ulcers, changes to glucose levels, muscle damage, low blood pressure, and changes in the heart rhythm [52].

NAM, which is also very soluble in water (approximately 1 g/mL), has the advantage of not causing skin flushing. At effective doses, its side effects are minimal, although at high doses it may also cause liver toxicity, nausea, vomiting, headache, fatigue, dizziness, and low platelets in the blood [53]. Moreover, a chronic and excessive intake of NAM may exacerbate motor symptoms of Parkinson’s disease [54].

A clinical trial, designed to assess the efficacy of a single-dose supplementation of NAM on NAD^+^ levels, showed that NAM intake within the daily tolerable upper level (200 mg) significantly increased the whole blood NAD^+^ 2–2.5-fold [55]. Olson et al. [56], using radiometric methods to characterize the uptake of [^14^C] NAM in human leukemic K-562 cells, demonstrated the binding of NAM to the plasma membrane, followed by its intracellular uptake and immediate synthesis to NAD^+^. However, it is uncertain how many cell types can take up significant amounts of NAM. Moreover, its efficacy decreases with age and stress [53], and inhibits different SIRT activities [57].

NMN is the phosphorylated form of NR. However, its NAD^+^-promoting activity mainly depends on the extracellular conversion of NMN to NR [58]. Then, NR enters the cells through nucleoside transporters and can be re-phosphorylated back to NMN through the reaction catalyzed by NRK [59]. Nevertheless, in rodents, it has been found that orally administered NMN quickly enters the cells of the small intestine through the Slc12a8 NMN transporter, and then is used to generate NAD^+^ [60]. This Slc12a8 carrier system appears approximately 100-fold more active in the small intestine than in other tissues, e.g., adipose cells and the brain [60]. Nevertheless, the preponderance of the evidence supports the idea that the main mechanism, whereby NMN increases intracellular NAD^+^ levels, relies on the extracellular metabolization of NMN to NR, which is then taken up by the cell and converted into NAD^+^. Considering all of this, it is critical to carefully take into account that findings in animal studies do not necessarily translate to humans. In practice, the first-in-humans clinical trial using NMN was recently published [61]. Specifically, 10 weeks of daily NMN supplementation (250 mg/day) in prediabetic women was shown to increase both circulating NAD^+^ levels and muscle insulin sensitivity [61]. NMN levels in the body were not reported.

Nicotinic acid has also been shown to both increase NAD^+^ levels in human cells through the nicotinic acid phosphoribosyltransferase-catalyzed reaction and to exert protection against oxidative stress [62].

Clinical research on the bioavailability of NR supplementation began in 2016 with a pilot study where a few people were given three single doses of NR. Each person completed each dose (100, 300, or 1000 mg) with a 7-day washout period between each one. The results showed that NR was bioavailable in supplement form and increased blood NAD^+^ when taken in aggregate (no single dose increased NAD^+^ levels significantly) [63]. In 2017, a randomized double-blind trial included 120 healthy subjects (60–80 years old) who were assigned to one of three groups in which each person completed eight weeks of daily supplementation: (1) a placebo group, (2) a dose of 250 mg NR + 50 mg pterostilbene (PT, a natural antioxidant found e.g., in blueberries), or (3) a dose of 500 mg NR + 100 mg PT [64]. The low-dose group showed a 40% increase in blood NAD^+^ compared to the placebo, while NAD^+^ levels in the higher-dose group increased by ~90%. No adverse side effects were observed, and it was concluded that the sustained use of NR safely increases NAD^+^ levels.

In 2019, a small group of 70 to 80-year-old men were given 1000 mg of NR supplementation per day in a randomized control crossover trial. The results showed that NR increased NAD^+^ in the skeletal muscle and also exerted anti-inflammatory properties [65]. In a work published that same year, Conze et al. reported the safety of the long-term (8 weeks) administration of NR (up to 1 g per day) to healthy overweight humans [66]. This study employed a dietary restriction designed to limit the amount of NAD^+^ precursors that could be obtained from the diet. The success of the dietary restriction is shown by the continued decline in NAD^+^ levels of the placebo group during the study. NR administration was also associated with an increase in blood NAD^+^ levels under these conditions [66].

Other approaches that could also be useful to increase NAD^+^ levels in vivo may include, analogs of NMN [67] or inhibitors of CD38 [68], PARP1 [69], SARM1 [70], and α-amino-β-carboxymuconate-ε-semialdehyde decarboxylase (ACMSD, a critical enzyme in de novo NAD+ biosynthesis; www.tespharma.com (accessed on 30 July 2021)).

### 3.2. Antioxidants

At present, only edaravone, a pyrazolone free-radical scavenger that may decrease oxidative damage, has been approved for ALS therapy. Its antioxidant effect has been known since 1994 and its impact on other diseases, such as ischemic stroke, was also investigated. However, it was not until 2006–2008 that its effectiveness was demonstrated in the SOD1^G93A^ mouse model [71,72]. In 2017, the FDA’s approval was based on the results of a double-blind, randomized, and placebo-controlled phase 3 clinical trial (NCT01492686) that evaluated its safety and effectiveness in treating ALS. After a 12-week observation period, participants were distributed into two groups. One group received 60 mg of edaravone (i.v.) per day for six months, while participants in the other group received a placebo. After the six-month-period of treatment, those who received edaravone experienced a 33% lower decline in the Revised Amyotrophic Lateral Sclerosis Functional Rating Scale (ALSFRS-R) score than those in the control group. Nevertheless, edaravone can cause hives, swelling, and shortness of breath in some people, due to anaphylactic reactions to sulfite-containing infusion components. Otherwise, it is rather well tolerated, although common side effects include walking problems, bruises, and headaches [73]. In a meta-analysis of published post-marketing clinical data, edaravone appeared effective in Asian countries, where its reported benefits on ALSFRS-R scores and lung capacity were similar to those seen in the clinical trials. The drug seems to have little clinical benefit in European countries, and the reason for this difference remains unclear [74].

In addition, other antioxidant molecules have also been considered for ALS therapy, i.e., (but not limited to) vitamin E, coenzyme Q10, melatonin, β-carotene, thiol donors, and natural polyphenols. However, data obtained in regular clinical trials are scarce and discouraging. The most promising of these antioxidant treatments, in the opinion of the authors of this review, are discussed below in more detail.

Vitamin E, in combination with the antiparkinsonian drug selegiline, was first assayed in an 18-month randomized treatment trial, but this long-term antioxidative treatment did not benefit patients with ALS [75]. However, in another double-blind placebo-controlled randomized trial, though vitamin E did not appear to affect the survival and neuromotor functions, patients receiving riluzole plus vitamin E remained in the milder clinical state of the disease for a longer period of time [76]. Nevertheless, at present, there is no evidence that vitamin E may benefit ALS patients once the disease has been diagnosed.

Coenzyme Q10, a mitochondrial cofactor known for its antioxidant properties, has a prolonged survival in mouse models of ALS and has a slowed functional decline in Parkinson’s disease. However, a phase II trial of Coenzyme Q10 in ALS did not render sufficient evidence to justify a phase III [77]. Nevertheless, a combined treatment of edaravone and coenzyme Q10 has been proposed [78].

In a small clinical safety study, chronic high-dose (300 mg/day) rectal melatonin was well tolerated during an observation period of up to 2 years. Circulating serum protein carbonyls (a marker of protein oxidation) were elevated in ALS patients, but were normalized to control values by melatonin treatment [79]. Moreover, two ALS cases, in which cocktails of supplements including melatonin were associated with a partial recovery of lost motor function, support further studies with melatonin, at least in a pilot trial [80].

High dietary intakes of β-carotene and lutein have been inversely associated with ALS risk [81]. Thus, the authors of this study suggest that the consumption of foods high in carotenoids may help to prevent or delay the onset of ALS, though we do not yet have evidence of the efficacy of this suggestion.

N-acetyl-L-cysteine (NAC), a direct thiol donor, was shown to improve the survival and neuromotor functions in the SOD1^G93A^ mouse model [82]. However, 50 mg of NAC/kg per day (S.C.) failed to significantly increase the survival or slow disease progression in a randomized, double-blind, and placebo-controlled clinical trial [83].

An ongoing 6-month open-label pilot trial is testing the effect of curcumin (a natural polyphenol responsible for turmeric’s yellow color) in ALS patients. This study is expected to be completed in 2021 (NCT04499963).

## 4. Potential Benefits of Combining NAD^+^ Precursors and Antioxidants in ALS Patients

EH301 (Elysium Health, NY, USA), containing two active ingredients (NR and PT), synergistically increases NAD^+^ levels and supports SIRT activity [84]. EH301 was efficacious in a placebo-controlled double-blind human pilot study in people with ALS. Following 4 months of treatment, a striking improvement was observed in all ALS-specific outcome measures in the EH301-treated group compared to the placebo [85], including:

- Revised ALS functional rating scale (ALSFRS-R) score: a 2.5-point improvement in the EH301 group, compared to a 5.5-point decline in the placebo group (the difference between the placebo and EH301 groups at the 4-month time-point = 6.1 points);
- Forced vital capacity (FVC): a 2.5% improvement in the EH301 group, compared to a 16.6% decline in the placebo group (the difference between the placebo and EH301 groups at the 4-month time-point = 19.4%);
- Medical Research Council (MRC) scale index: a 17-point improvement in the EH301 group, compared to an 11-point decline in the placebo group (the difference between the placebo and EH301 groups at the 4-month time-point = 23 points).

These results were accompanied by significant improvements in the muscle activity within the triceps, measured by electromyography. In this trial, all participants were also taking riluzole. No side effects attributed to the investigational product were observed in any study participants.

Importantly, patients were also evaluated after completing the first year of treatment. Relative to the baseline, no significant deterioration in the ALSFRS-R score or muscle function was observed, measured using the MRC grading scale. In addition, five of the eight muscle groups investigated using electromyography did not show deterioration. However, a 11.5% reduction in FVC was detected, suggesting a decline in pulmonary function between the baseline and 1 year. Importantly, this reduction in FVC after 1 year was smaller than the reduction in FVC observed in the placebo group at 4 months (11.5% mean reduction vs. 16.7%), indicating a prolonged slowing of disease progression [85].

The limitations of this pilot study include the small number of patients (only 20 participants finished the trial), the lack of biomarkers, and whether a couple of dropouts in the EH301 group were related to the treatment or not. 

Despite these limitations, this study is the basis for a phase 2 human study currently underway in Norway, dubbed the NO-ALS Study (NCT04562831). This trial will examine these preliminary results in a much larger population of ALS patients in order to evaluate its true effectiveness.

**Figure 1:**
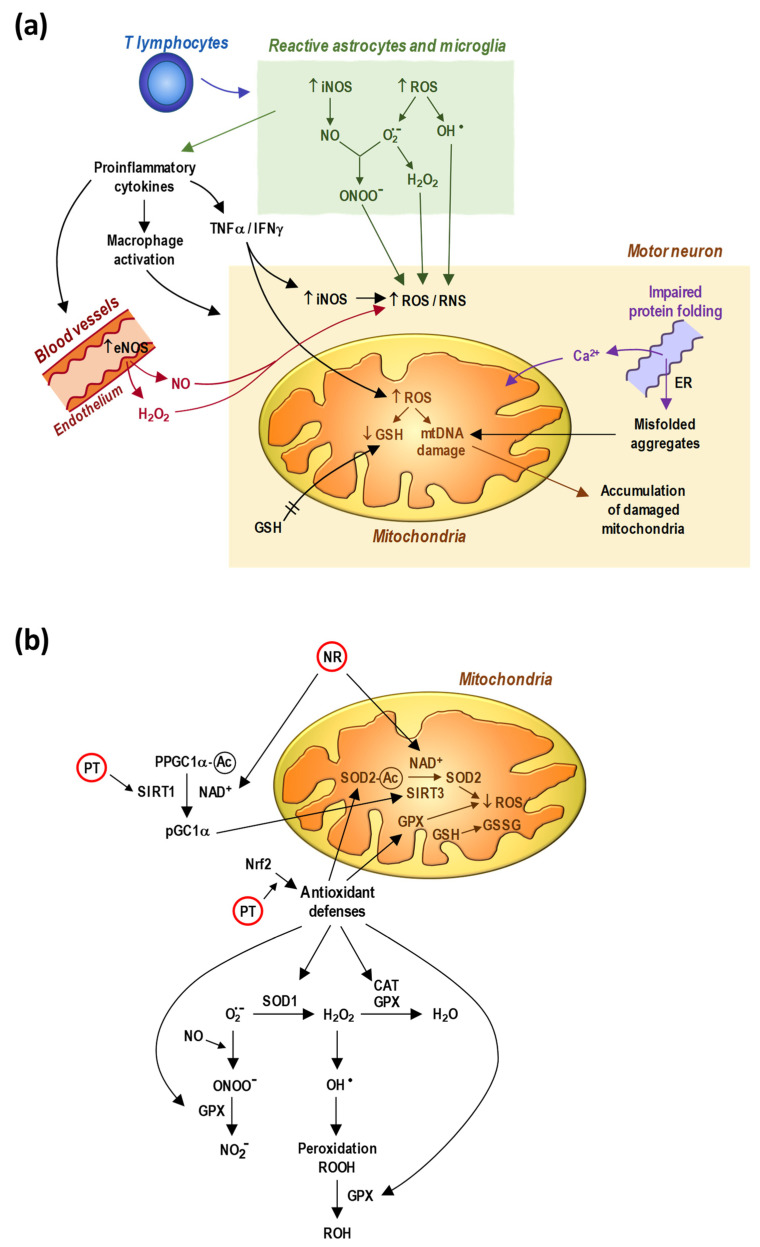
The association of NAD^+^ boosters and antioxidants may interfere in the pathophysiology of ALS and be beneficial for the protection of motor neurons. Reactive oxygen and nitrogen species (ROS and RNS) can be generated by reactive glial cells, endothelial capillary cells, and the motor neurons. Recurring stress could generate a vicious cycle of accumulated damages until reaching a limit of no return that leads to the inevitable death of MNs: (**a**) cellular interactions that condition mitochondrial damage associated with the activation of cell death; (**b**) in the molecular interactions displayed, nicotinamide riboside is used as an example of NAD^+^ promoter, and pterostilbene as an antioxidant molecule capable of inducing SIRT1 and Nrf2 activation. Furthermore, NAD^+^ kinase converts NAD^+^ into NADP^+^, and the pentose phosphate pathway generates NADPH, essential for the reduction of GSSG to GSH catalyzed by the glutathione reductase in the presence of flavin adenine dinucleotide [86]. On the other hand, the membrane-bound NADPH oxidases catalyze the production of O_2_^.−^ by transferring one electron from NADPH to O_2_ [87]. Both are opposite effects that should be taken into account in the design of a well-balanced therapy. eNOS, endothelial nitric oxide synthase; iNOS, inducible nitric oxide synthase; SIRT, sirtuin; SOD, superoxide dismutase; CAT, catalase; GPX, glutathione peroxidase; O_2_^.−^, superoxide anion; ONOO^−^, peroxynitrite; NO_2_^−^; nitrite; H_2_O_2_, hydrogen peroxide; ^.^OH, hydroxyl radicals; ROOH, peroxides; GSH, glutathione; GSSG, glutathione disulfide; mtDNA, mitochondrial DNA; ER, endoplasmic reticulum; Nrf2, nuclear factor erythroid 2-related factor 2; pPGC1α, phosphorylated peroxisome proliferator-activated receptor gamma coactivator 1-α; NR, nicotinamide riboside; PT, pterostilbene.

Regardless of the results of these trials, what is indeed relevant is the possibility that the combination of a NAD^+^ booster plus an antioxidant molecule could render benefits in the treatment of ALS. Figure 1 displays a scheme showing how an increase in NAD^+^ levels and the action of specific antioxidant molecules could interfere with the mechanisms leading to mitochondrial damage and MN death. Reactive species derived from oxygen and nitrogen are generated by microglia and astrocytes, by vascular endothelial cells, and by the MNs themselves. In this mechanism, proinflammatory cytokines, such as TNFα and IFNγ, further enhance the generation of ROS and RNS within the MNs and the damage of mitochondrial functions. Thiol oxidation favors both the release of Ca^2+^ ions from the endoplasmic reticulum and the formation of misfolded protein aggregates, which also favor mitochondrial malfunction (see Figure 1a). PT promotes the antioxidant defense of the MNs, whereas NR favors the formation of NAD^+^ and the SIRT-dependent mechanisms of that defense (see Figure 1b).

## 5. A Combination Therapy Strategy for ALS

Until now the most common strategy in clinical trials against ALS has been to test individual molecules. However, ALS is a complex pathology with multiple cellular and signaling interactions; a similar problem to the one that oncologists grapple with when treating cancers. In this regard, years ago, oncologists realized that the combined use of different treatment strategies was the right way to achieve the best possible results. It is the opinion of the authors of this review that ALS treatment should follow the same (multi-target) strategy. 

Emerging evidences suggest a key role of NAD^+^ depletion and impairment of NAD^+^-dependent pathways in different neurodegenerative diseases, i.e., (but not limited to) Alzheimer’s, Parkinson’s, and Huntington diseases, as well as (as discussed above) ALS (see Lautrup 2019 for a recent comprehensive review) [17]. In addition, antioxidants have also been considered a potential approach to slow the progression and limit the extent of neuronal cell loss in neurodegenerative diseases, and also to decrease the glia-mediated inflammation [88,89]. At present, there is a lack of clinical-trial-based evidence supporting a benefit of NAD^+^ promoters or antioxidants in ALS. However, the pilot study combining NR and PT supports this approach with the strong possibility of finding real benefits. In this regard, and based on the above discussion, NR seems to be the best NAD^+^ promoter available. Alternatively, methods to increase physiological antioxidants (such as GSH) or to activate Nrf2 (a master regulator of the cell antioxidant defense) could be alternatives to the use of direct antioxidant molecules. For instance, N-acetyl-cysteine has been shown to promote GSH synthesis by delivering Cys, which is rate-limiting for the synthesis of the tripeptide [84]. In addition, different electrophilic compounds or protein–protein inhibitors of the Keap1–Nrf2 system could work as Nrf2 activators [90]. 

In order to further complement this therapeutic strategy, we can propose two additional molecules that could add benefits if applied in combination. First, neuroinflammation has been well documented as a main mechanism involved in the pathophysiology of ALS. As displayed in Figure 1, inflammatory cytokines are part of the mechanism of the disease. Hence, it makes sense to consider anti-inflammatory drugs for the combination. For instance, cromolyn sodium (an anti-inflammatory agent that prevents mast cell activation and degranulation by inhibiting chloride transport and protein kinase C) significantly delays ALS symptom onset and disease-associated neuromotor problems in the SOD1^G93A^ model compared with untreated control mice [91]. Cromolyn sodium-treated mice showed a significant decrease in the levels of several proinflammatory mediators in both the spinal cord (CXCL1 and TNFα) and blood (IL2, IL6, and IL10) [91]. Besides, phosphodiesterase (PDE) activity inhibitors are another interesting anti-inflammatory strategy for ALS [92]. One of its inhibitors, ibudilast (an inhibitor of macrophage migration inhibitory factor and phosphodiesterases 3, 4, 10, and 11), is currently being assayed in ALS patients (NCT04057898, COMBAT-ALS).

Second, in ALS it is also known that glucose transport and metabolism can be progressively impaired in the MNs (as recently analyzed in Tefera 2021) [93]. Thus, a modified ketogenic diet (e.g., enriched in mid-chain fatty acids as an alternative source of energy) could also complement a combined therapy [94,95].

## 6. Conclusions

The discussion presented in this review offers alternatives for their application in ALS therapy. Nevertheless, we think that the most important point of this review is the suggestion to seriously consider the potential benefits of combined strategies targeting different pathways/molecules at the same time. In this regard, platform-based trials where multiple drugs can be tested in tandem against one placebo group could be very helpful. Considering all of this, there is an unmet need to validate biomarkers in order to differentiate between responders and non-responders.

## Abbreviations

ALS: amyotrophic lateral sclerosis; MN: motor neuron; SALS: sporadic ALS; FALS: familial ALS; ROS: reactive oxygen species; SIRT: sirtuin; PARP1: poly(ADP-ribose) polymerase 1; NAM: nicotinamide; NR: nicotinamide riboside; NRK: nicotinamide riboside kinase; NNMAT1: nicotinamide mononucleotide adenylyl transferase 1; SARM1: sterile α and TIR motif–containing 1; SOD: superoxide dismutase; GSH: glutathione; GSSG: glutathione disulfide; NA: niacin; NMN: nicotinamide mononucleotide; PT: pterostilbene; ACMSD: α-amino-β-carboxymuconate-ε-semialdehyde decarboxylase; ALSFRS-R: revised amyotrophic lateral sclerosis functional rating scale; NAC: N-acetyl-L-cysteine; FVC: forced vital capacity; MRC: Medical Research Council; eNOS: endothelial nitric oxide synthase; iNOS: inducible nitric oxide synthase; ROOH: peroxide; mtDNA: mitochondrial DNA; ER: endoplasmic reticulum; Nrf2: nuclear factor erythroid 2-related factor 2; pPGC1α: phosphorylated peroxisome proliferator-activated receptor gamma coactivator 1-α; PDE: phosphodiesterase.
